# A Study on the Acceptance of Smart Cane Technology Among Chinese Older Adults

**DOI:** 10.3390/healthcare13222934

**Published:** 2025-11-16

**Authors:** Yibing Chen, Yi An, Zihao Chen, Dingbang Luh, Tiansheng Xia

**Affiliations:** School of Art & Design, Guangdong University of Technology, Guangzhou 510090, China

**Keywords:** smart canes, technology acceptance, older adults, extended TAM, mediating effect

## Abstract

**Background:** Although smart products improve older adults’ quality of life, their acceptance and usage of smart assistive devices remain relatively low, and the influencing factors remain unclear. **Methods**: This study takes canes, which are commonly used by older adults, as the research object. To explore older adults’ acceptance of smart canes and the influencing factors, we constructed an integrated framework based on the extended Technology Acceptance Model (TAM), which incorporates multiple variables: Perceived Usefulness (PU), Perceived Ease of Use (PEOU), Attitude (ATT), Social Influence (SI), Safety Trust (ST), Self-Efficacy (SE), and Behavioral Intention (BI). A questionnaire survey was conducted to collect 232 valid responses, and structural equation modeling (SEM) was employed for data analysis. **Results**: The results indicate that factors including PU, PEOU, and SI have significant impacts on older adults’ ATT towards using smart canes, their SE, and BI. Among these factors, ATT and SE play a mediating role between PU, PEOU, SI, and BI in using smart canes. **Conclusions**: The practical implications of the current results are discussed with the aim of providing empirical evidence for the development and application of smart assistive devices for older adults.

## 1. Introduction

By 2020, individuals aged 60 and above constituted 18.7% of China’s total population. Projections indicate that by 2050, older adults’ population in China will reach approximately 454 million, making up 33% of the total population [1]. Due to declining cognitive and motor functions, older adults often struggle to complete daily tasks independently, increasing their dependence on others [2]. Many older adults experience issues such as muscle weakness and increased fatigue [3]. The loss of mobility not only deteriorates their physical health but also negatively impacts their psychological, social, and economic wellbeing. Against the backdrop of global aging, enhancing self-management capabilities during illness has become a crucial issue [1], and canes, as mobility aids for older adults, are a key product for improving self-management capabilities. However, traditional canes have limited effectiveness in meeting older adults’ daily mobility needs [4], while the use of smart assistive technologies may reduce older adults’ reliance on family members or caregivers [5]. Smart canes are innovative mobility aids that integrate traditional physical support with modern intelligent technologies. Each smart cane consists of a main body and intelligent technologies installed on the cane, including an input module, an environmental data collection module, a main control module, a feedback module, and a drive module [6]. Compared with traditional canes (which only provide basic balance support), smart canes can proactively warn of hazardous situations and send location-linked distress signals in emergencies. They play a crucial role in enhancing the mobility, safety, and independence of older adults [7].

The development of smart technologies has driven the emergence of smart assistive devices, which are beneficial for restoring older adults’ walking ability and alleviating the burden on healthcare professionals [3]. By integrating multiple technologies, smart assistive devices can meet older adults’ daily needs. Therefore, older adults’ willingness to use these devices and their perception of new technologies are critical to determining adoption [8]. Nevertheless, studies have shown that older adults do not fully accept modern technology, and their usage rate of smart devices and other technologies is lower than that of young people [9]. Olson et al. revealed that compared with young people, older adults tend to use older technologies rather than the latest ones in fields such as communication, customer service, healthcare activities, and home systems [10]. Verdegem and De Marez pointed out that due to insufficient attention to user acceptance, technological innovations often fail to be fully utilized. Hence, it is particularly important to investigate older adults’ views and influencing factors regarding smart canes that apply new technologies [11].

To explore the acceptance of smart canes among older adults, we conducted a relevant study using the Technology Acceptance Model (TAM). Proposed by Davis, the TAM is an important theoretical model for measuring users’ willingness to use new technologies. It explains the interrelationship between external variables affecting users’ acceptance of technology and factors influencing actual usage behavior [12]. It has developed into a key model for understanding the potential predictors of human behavior in accepting or rejecting technology [13,14]. Previous studies have demonstrated the effectiveness of the TAM in multiple fields and disciplines [15], and it has also been used to examine older adults’ acceptance of new technologies in areas such as healthcare and assistive technology, social networks, online shopping, the Internet, computers, online public services, and entertainment [16]. To enhance the model’s predictive ability and applicability in different fields, researchers have introduced new variables to extend the TAM [13]. For example, Zin used the extended TAM to explore the acceptance of digital health wearable technologies among older adults in South Korea and found that Perceived Usefulness (PU), Perceived Ease of Use (PEOU), and facilitating conditions have significant impacts on older adults’ usage Attitude (ATT) [8]. Etemad-Sajadi and Gomes Dos Santos extended the TAM by introducing variables such as social presence, trust, and perceived intrusiveness in their study to investigate older adults’ acceptance of home-based connected health technologies [17]. In addition, researchers found that PU, compatibility, facilitating conditions, and self-reported health status have significant and positive effects on older adults’ intention to use smart wearable systems [18].

Although some studies have investigated older adults’ views on healthcare and technology acceptance [19,20], studies focusing on intelligent assistive products like smart canes are relatively scarce. Most existing studies concentrate on the general application of the TAM, and the selection of variables fails to fully consider the unique attributes of smart canes and the specific needs of older adults. Meanwhile, the research perspective is relatively narrow, lacking studies that analyze the factors influencing older adults’ acceptance of smart canes technology from multiple dimensions. Furthermore, when emphasizing that technological factors affect users’ Behavioral Intention (BI) to adopt new technologies, researchers often overlook the impacts of users’ capabilities, resources, and social environmental factors [21]. As a group that includes many people with disabilities, older adults’ perception of their own capabilities and Self-Efficacy (SE) often plays an important role in their decision-making [22]. Moreover, older adults usually have more profound and long-lasting emotional states, greater loneliness than young people, and greater susceptibility to social influences (SI), with higher requirements for Safety Trust (ST) [23]. Therefore, it is of great significance to explore the influencing factors of older adults’ acceptance of smart canes through the extended Technology Acceptance Model that incorporates three variables: SE, SI, and ST.

## 2. Research Variables and Hypothesis Formulation

### 2.1. Behavioral Intention (BI) and Attitude (ATT)

BI is defined as “the degree to which an individual formulates a conscious plan to perform or not perform certain specific behaviors in the future”. Studies have proven that an individual’s BI to use technology has a positive impact on their actual use of the technology [24]. Therefore, many studies focus on the BI to use technology to predict actual usage and technology acceptance [18,25]. The ATT factor is defined as “an individual’s feelings or evaluations towards using a certain technology” [24]. Multiple studies have shown that individuals with a positive ATT towards a certain technology will have a strong willingness to use it [26,27,28]. Therefore, we propose the following hypothesis:

**H1.**  *The ATT towards using smart canes can significantly and positively predict the BI to use them*.

### 2.2. Social Influence (SI)

SI is defined as the degree to which other people (family members, friends, peers, and caregivers) believe (positively or negatively) that something will affect an individual’s use of new technologies [24,29]. Individuals may change their behaviors based on the suggestions of important family members or friends to strengthen these social relationships [30,31]. SI may come from family members, peers, and professional caregivers of older adults. Previous studies have shown that SI is important in determining the use of technology for elderly care [32,33], and that it is a crucial determinant of older adults’ usage ATT and BI [24,34]. For example, a study by Kijsanayotin found that SI is an important factor in determining the use of electronic medical records and health information technology [35]. A study by Chen and Chan further indicated that SI can indirectly affect usage willingness through ATT [36]. Therefore, the current study proposes the following hypotheses:

**H2.**  *SI can significantly and positively predict older adults’ BI to use smart canes*.

**H3.1.**  *SI can significantly and positively predict older adults’ ATT towards using smart canes*.

**H3.2.**  *ATT plays a mediating role between SI and BI to use smart canes*.

### 2.3. Safety Trust (ST)

ST refers to the degree to which an individual considers the information and services provided by a specific technology to be reliable [27,37]. Previous studies have found that information credibility is crucial for the success of health-related portals for health management [27]. The more credible health information is, the greater the probability that it will be successfully utilized [38,39]. For instance, Zhang et al. reported that trust is the most critical factor in promoting a positive ATT towards autonomous vehicles [28]. Kamal et al. studied the factors influencing patients’ willingness to use telemedicine and found that trust is one of the most important determinants in this regard [40]. In the current study, ST reflects older adults’ level of trust in the safety of using smart canes, including information security and usage safety, which may affect their ATT towards using them. Therefore, this study proposes the following hypotheses:

**H4.**  *ST can significantly and positively predict older adults’ BI to use smart canes*.

**H5.1.**  *ST can significantly and positively predict older adults’ ATT towards using smart canes*.

**H5.2.**  *ATT plays a mediating role between ST and BI to use smart canes*.

### 2.4. Self-Efficacy (SE)

SE is rooted in social cognitive theory [41] and refers to an individual’s belief in their ability to perform a task. Individuals with high SE are more likely to view difficult tasks as needing to be mastered rather than avoided [42]. The social cognitive theory proposed by Cleary and Kitsantas suggests that an individual’s belief in their own abilities directly drives their behavior [43]. For example, SE can predict medication adherence in elderly patients with hypertension and regulate the relationship between depression and medication adherence [44]. Regarding using new technologies, SE often means an individual’s cognition that they have sufficient and accurate abilities and skills to achieve success [45]. Since some older adults have a certain degree of disability, and functional disability can easily lead to psychological insecurity, this has a significant impact on their decision-making in daily life [46]. In addition, a survey conducted by Chen and Chan on the acceptance of technology among Chinese older adults in Hong Kong found that SE can indirectly affect usage willingness through ATT [36]. Based on these studies, we propose the following hypotheses:

**H6.1.**  *Older adults’ SE can significantly and positively predict their ATT towards using smart canes*.

**H6.2.**  *ATT plays a mediating role between SE and BI*.

### 2.5. Perceived Ease of Use (PEOU) and Perceived Usefulness (PU)

Perceived ease of use is defined as the degree to which an individual believes that using a technology demands minimal effort [47]. In this study, it refers to the degree of difficulty that older adults perceive in using smart canes. The TAM states that PU and PEOU are the main factors affecting usage ATT. PU is defined as the degree to which an individual believes that using a specific technology will improve their quality of life [12]. In this study, it refers to the degree to which older adults believe that using smart canes can bring convenience to their lives, improve their quality of life, and enhance their health management. Studies consistently show that PU and PEOU significantly affect an individual’s ATT and BI towards using technology [48,49]. In addition, a study by Jen and Hung on older adults’ technology acceptance of mobile health services found that PU and PEOU can indirectly affect older adults’ BI through ATT [47]. Therefore, hypotheses H7 and H8 are proposed:

**H7.1.**  *PEOU can significantly and positively predict the ATT towards using smart canes*.

**H7.2.**  *ATT plays a mediating role between PEOU and BI to use smart canes*.

**H8.1.**  *PU can significantly and positively predict the ATT towards using smart canes*.

**H8.2.**  *Older adults’ ATT towards using smart canes plays a mediating role between PU and BI to use smart canes*.

Considering that SE is an important factor affecting PEOU and PU, older adults’ understanding and perception of their own knowledge and skills determine how they evaluate the ease of use and usefulness of technical tools [50]. Existing studies have found that the PEOU and PU of a product have an impact on users’ SE [36]. Therefore, combined with hypothesis H6, we propose hypotheses H9 and H10:

**H9.1.**  *PEOU can significantly and positively predict SE*.

**H9.2.**  *SE plays a mediating role between PEOU and usage ATT*.

**H9.3.**  *SE and usage ATT play a chain mediating role between PEOU and BI to use smart canes*.

**H10.1.**  *PU can significantly and positively predict SE*.

**H10.2.**  *SE plays a mediating role between PU and usage ATT*.

**H10.3.**  *SE and usage ATT play a chain mediating role between PU and BI to use smart canes*.

**Figure 1:**
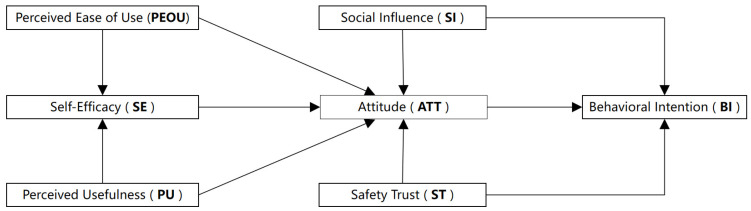
Research framework.

To sum up, to systematically study older adults’ acceptance of smart canes and its influencing factors, we construct an extended research framework based on the TAM by introducing three variables: SE, SI, and ST (Figure 1).

## 3. Materials and Methods

### 3.1. Participants

A convenience sampling method was adopted, and questionnaires were distributed to participants in urban communities and suburban activity centers. On-site staff provided guidance to ensure older adults could complete the questionnaires correctly. To help participants fully understand smart canes’ core functions and avoid response deviations caused by limited knowledge of smart technologies, staff specifically explained the key functional modules of smart canes to each participant. These functions include built-in multi-axis sensors for real-time posture monitoring, GPS positioning and tracking to support family members in checking location, automatic fall detection and one-click emergency call, and data linkage with health management apps to synchronize walking distance, heart rate and other health indicators. For participants who had doubts about functional details—such as how the fall detection function distinguishes between normal sitting and accidental falls—staff provided patient answers and demonstrated the operation process using a smart cane prototype when conditions permitted, ensuring that each participant could intuitively recognize the practical value and usage method of smart canes.

Eventually, a total of 250 questionnaires were distributed, and 232 valid questionnaires were recovered, resulting in an effective recovery rate of 92.80%. All 232 participants were recruited from Guangzhou, Guangdong Province, with 68.1% (n = 158) from urban communities, 31.9% (n = 74) from suburban elderly activity centers. All participants provided written informed consent, and this study was approved by the Academic Ethics Review Committee of Guangdong University of Technology (Approval No. GDUTXS2024128). We gave umbrellas to participants as a reward for their participation in the study. The demographic distribution of the survey sample is presented in Table 1.

### 3.2. Research Instruments

The questionnaire was designed based on existing mature scales identified through a literature review of technology acceptance research. Several measurement items were modified to better suit the objectives of this study. The questionnaire consists of three sections. The first section provides a brief introduction to smart canes and related examples. The second section collects participants’ demographic information (e.g., age, gender, physical health status, and experience in using smart devices). The third section includes questions related to the acceptance of smart canes, covering measurement items for variables such as PU, PEOU, SI, ST, ATT, BI, and SE. For each variable, multiple specific questions were designed to comprehensively understand older adults’ cognition and usage willingness towards smart canes. A 5-point Likert scale was used for measurement, where “1” represents “strongly disagree” and “5” represents “strongly agree”. For example, to measure PU, items such as “Using smart canes will help me manage my health”, “I believe that using smart canes will make my daily life more convenient”, and “Using smart canes will improve my quality of life” were set. Through older adults’ self-reported responses, the degree of their PU of smart canes was quantified. Similar measurement methods were adopted for other variables to ensure the quantifiability and comparability of the data. The questionnaire items and their sources are presented in Table 2.

## 4. Results

### 4.1. Measurement Model

The first stage involved evaluating the measurement model [52]. We used AMOS 24.0 to examine the internal consistency, reliability, and convergent validity of the constructs in the proposed model. The results showed that the confirmatory factor analysis (CFA) fit indices of the measurement model were well-fitted: χ/df = 1.746, CFI = 0.945, TLI = 0.932, IFI = 0.944, RMSEA = 0.057, and SRMR = 0.042. For internal consistency, the factor loadings of all measurement items were higher than 0.6, meeting the standard level. In addition, the Cronbach’s α coefficient, composite reliability (CR), and average variance extracted (AVE) of the seven latent variables all reached the standard levels, indicating that the measurement model had acceptable reliability and convergent validity. The specific values are shown in Table 3.

Subsequently, we used the software SmartPLS 4.0 to verify the discriminant validity of the model, adopting the heterotrait–monotrait (HTMT) ratio method to assess the discriminant validity of each construct. The HTMT ratio has been proven to be a more rigorous and effective method for testing discriminant validity [53], and ideally, the HTMT value should be below 0.85 [54]. According to the results in Table 4, discriminant validity can be established based on the HTMT method, and all values in the table are less than 0.85, indicating sufficient discriminant validity. Combining the results of the above tests, it is proven that the scale in this study has good reliability, validity, and discriminant validity.

### 4.2. Structural Model and Hypothesis Testing

In the second stage, we focused on evaluating the structural model. Structural equation modeling (SEM) was constructed using AMOS 28.0, and the 232 valid datasets collected from the questionnaires were imported. First, we tested the goodness-of-fit of SEM. The hypothetical relationships of the research model were established using SEM (see Figure 1 for the research model). The goodness-of-fit indices of SEM were good: χ/df = 1.942, CFI = 0.926, TLI = 0.914, IFI = 0.927, and RMSEA = 0.064. The SEM was used to model and evaluate the hypothetical relationships of the research model, and the results are shown in Table 5.

**Figure 2:**
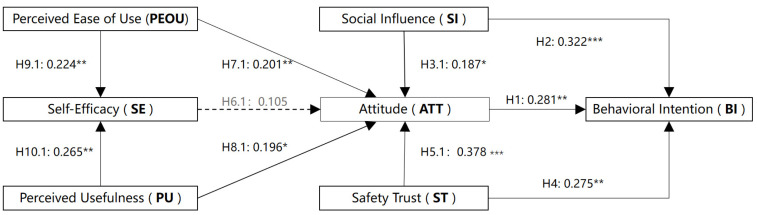
The result of the direct path. Note: * *p* < 0.05, ** *p* < 0.01, *** *p* < 0.001.

ATT (*β* = 0.281, *p* < 0.01) had a significant positive impact on BI, supporting H1; SI (*β* = 0.322, *p* < 0.001) had a significant positive effect on BI, supporting H2; SI (*β* = 0.187, *p* < 0.05) positively and significantly affected ATT, supporting H3; ST (*β* = 0.275, *p* < 0.001) had a significant positive effect on BI, supporting H4; the path from ST (*β* = 0.378, *p* < 0.01) to ATT was significant, supporting H5; however, the path from SE to ATT was not significant, so hypothesis H6 was not supported; PEOU (*β* = 0.201, *p* < 0.01) had a significant positive impact on ATT, supporting H7; PU (*β* = 0.196, *p* < 0.05) had a positive impact on ATT, supporting H8; both PEOU (*β* = 0.224, *p* < 0.01) and PU (*β* = 0.265, *p* < 0.01) had significant positive impacts on SE, supporting H9 and H10. According to the results, among the 10 direct paths related to users’ intention to use smart canes in the model, 9 were verified (Figure 2).

### 4.3. Mediating Effect Analysis

Subsequently, we further analyzed the mediating roles of SE and ATT between PEOU, PU, ST, SI, and BI. Therefore, using AMOS 28.0, the bootstrap method was applied to analyze the mediating effects of the current model, with 2000 resamplings conducted at a 95% confidence level. The analysis results showed that five mediating paths were valid (Table 6). Combining the results of the mediating effect analysis, it was found that ATT played a significant mediating role between ST and BI; SE played a significant mediating role between PEOU, PU, and ATT; and SE and ATT played a significant chain mediating role between PEOU, PU, and BI.

## 5. Discussion

### 5.1. Analysis of Structural Model and Hypothesis Testing Results

In this study, we found that older adults’ positive ATT towards smart canes significantly and positively predicts their BI to use them (H1 is supported). This result is consistent with the core view of the TAM. Davis et al. pointed out in the original TAM theory that an individual’s ATT towards technology is a core mediating variable affecting their usage intention: the more positive the ATT, the stronger the BI. In addition, SI has a significant positive effect on both BI and ATT (H2 and H3.1 are supported), but the mediating effect of ATT between SI and BI is not significant (H3.2 is not supported).

The predictive role of SI is closely related to the social dependence characteristics of older adults. Doekhie et al. found that in the decision-making process of elderly patients regarding healthcare-related technologies, the opinions of “trusted groups” such as family members and medical staff carry more weight than those of the control group, and their decision-making is essentially a manifestation of social identity—where older adults align their choices with the evaluations of trusted others—which explains why SI directly shapes their BI [55]. Therefore, when their children or doctors recommend smart canes, older adults directly perceive them as “reliable choices” and form usage intentions without the need for ATT as an intermediary. This phenomenon reflects older adults’ tendency to rely on external social evaluations when making decisions about new technologies, especially health-related ones, which helps explain why SI can directly predict BI.

### 5.2. Mechanism of the Relationship Between Behavioral Intention, Attitude, and Safety Trust

ST has a significant positive predictive effect on both BI and ATT, and the mediating effect of ATT between the two is significant (H4 and H5 are supported). This indicates that ST is the “core threshold” for older adults to accept smart canes. This result is highly consistent with the risk-aversion characteristics of older adults and the product attributes of smart canes. The importance of ST stems from the particularity of health-related technologies. A study by Bluethmann et al. on elderly cancer patients showed that older adults’ concerns about the safety of smart canes are a key factor affecting their acceptance, and respondents listed “usage safety” as their primary concern [56].

In a study on the credibility of safety information, Gao et al. found that participants’ level of engagement with an issue affects their perception of credibility [38]. For older adults, smart canes are not only tools to improve the quality of life but also “safety guarantees” for daily travel. When they believe that the safety performance of smart canes is reliable, they will form a positive ATT of “this product is beneficial to me and risk-free”, which, in turn, strengthens their usage intention. This “ST→ATT→BI” path fully reflects older adults’ priority of safety needs in the acceptance of health-assistive technologies.

### 5.3. Mechanism of the Relationship Between Perceived Ease of Use/Perceived Usefulness, Self-Efficacy, Attitude, and Behavioral Intention

This study found that the higher older adults’ PEOU or PU of smart canes, the more positive their usage ATT (H7.1 and H8.1 are supported), and the stronger their SE (H9.1 and H10.1 are supported). Additionally, SE plays a mediating role between PEOU, PU, and usage ATT (H9.2 and H10.2 are supported). This indicates that PU and PEOU have positive effects on older adults’ ATT towards using smart canes and their SE, consistent with previous studies [48,49].

However, we also found that SE cannot directly predict older adults’ ATT towards using smart canes (H6.1 unsupported), and ATT does not play a significant mediating role between SE, PEOU, PU, and BI (H6.2, H7.2, H8.2 unsupported). We speculate that this is mainly due to the unique characteristics of older adults and their insufficient understanding of smart canes. Due to their distinctive physical and psychological traits, older adult users may need to devote more effort and time to familiarize themselves with information technology [57]. The trust of older adults plays a more prominent role in the decision-making process concerning health-related assistive technologies [58]. Furthermore, older adults influenced by collectivism rely more on “external recognition” from family members, communities, or medical staff, rather than forming ATT solely based on their own usage capabilities. Studies have indicated that positive ATT can be translated into effective practices when supported by sufficient knowledge [59]. However, we found that older adults lack adequate technical understanding of smart canes, which may be the primary reason why ATT fails to function as a predictor.

As Bluethmann et al. noted, smart canes are not only “tools to improve the quality of life” but also “key equipment to ensure travel safety”, and their “safety attribute” takes priority over ordinary technical products [56]. A study by Wallin et al. on the occupational SE of elderly engineers found that due to the unique characteristics of the older adult group, their SE is affected by multiple factors [46]. For older adults, smart canes are not simply “ability-accessible” products; they involve travel safety and health management, rather than just focusing on their own usage abilities. Therefore, even if older adults believe they have the ability to use smart canes, they may still form a negative ATT if they worry about falls caused by operational errors or redundant and useless complex functions.

This study also found that SE and ATT play a chain mediating role between PEOU and BI (H9.3 and H10.3 are supported). This essentially reflects the activation of older adults’ progressive decision-making chain of “ability confirmation → attitude enhancement → behavior transformation”, a chain that compensates for the deficiency of the single path of “perceived attribute → attitude → behavioral intention” and conforms to the cognitive characteristics of older adults and the safety requirements of smart canes. K. Chen and Chan also found that PEOU and PU can synergistically affect usage behavior by influencing ATT and other constructs [9]. Specifically, when older adults believe that smart canes have practical functions such as health monitoring, they will take the initiative to learn how to use them. After mastering the usage methods, their SE is enhanced; this sense of SE makes them develop a positive ATT towards the product and promotes their usage intention.

## 6. Conclusions

Based on the extended TAM, this study introduced three key variables—SE, SI, and ST—to construct an integrated research framework. By analyzing data from 232 questionnaires using structural equation modeling, this study systematically explored the technology acceptance of smart canes among Chinese older adults and the influencing factors. The results showed that nine of the hypotheses regarding direct paths were supported, while only the hypothesis that SE directly predicts ATT was not supported. Additionally, five significant mediating paths were identified.

In-depth exploration of older adults’ acceptance of smart cane technology can provide a scientific basis for optimizing the design of smart canes. By understanding the needs and preferences of older adults, enterprises can improve product functions, enhance product ease of use, and develop smart canes that better align with their usage habits. The research findings can also assist in formulating effective marketing strategies to increase the penetration rate of such products among older adults, thereby improving their quality of life and addressing the issue of population aging. From the perspective of design and development, based on the verified paths of “ST→ATT→BI” and “PEOU→SE →ATT”, the shape of smart canes can be designed to match the hand dexterity of older adults. Meanwhile, their operation process should be simplified to reduce the cognitive load of older adults. From the perspective of policy promotion, in accordance with the research conclusion that SI directly predicts BI, local governments can organize “Smart Cane Family Experience Workshops” in community elderly activity centers, invite adult children and caregivers to participate in product usage demonstrations, and include smart canes that meet the aforementioned usability standards in local elderly care service procurement lists, so as to increase product penetration rate. By integrating the TAM and the health needs of older adults, this study provides a new perspective for the research on the “technology–human–health management” synergistic mechanism in the field of health informatics and further enriches the theoretical applications and practical cases of health informatics in the field of elderly-specific assistive devices.

This study also has several limitations that need to be addressed. All participants in this study were recruited from Guangzhou, Guangdong Province, resulting in an obvious regional concentration. Thus, the findings cannot be directly generalized to older adult populations in regions with cultural differences. This study used a cross-sectional survey design, which cannot verify the long-term causal relationships between the variables. Future studies may use a longitudinal design; for example, long-term follow-up surveys can be conducted to observe the dynamic evolution of the relationships between variables. For the unsupported hypotheses, future studies can further explore the underlying influencing mechanisms to provide more in-depth theoretical support for the development of smart canes.

## Figures and Tables

**Table 1:** Demographic characteristics (N = 232).

	Item	Number	Percentage (%)
Gender	Male	104	44.83
	Female	128	55.17
Education Level	Elementary school and below	47	20.26
	Secondary school education	116	50.00
	College degree and above	69	29.74
Region	Urban communities	158	68.10
	Suburban activity centers	74	31.90
Age	55–64 years	49	20.12
	65–74 years	96	41.38
	75 years and above	87	37.50
Health status	Good	62	26.72
	General	155	66.81
	Poor	15	6.47
Usage Experience	Never used	146	62.93
	Used	86	37.07

**Table 2:** Construct of the questionnaire.

Construct	Item	Reference
SE1	I am confident that I can use a smart cane effectively.	[24]
SE2	I can go out independently with the help of a smart cane if I have just the instruction manual for assistance.	
SE3	I think I can solve the problems I encounter when using a smart cane.	
ATT1	Using a smart cane is a good idea.	[12]
ATT2	Using a smart cane gives me the opportunity to gain healthy recognition.	
ATT3	The smart cane allows me to have more common topics with others and increase communication opportunities.	
SI1	People who are important to me think that I should use a smart cane.	[24]
SI2	People who influence my behavior think that I should use a smart cane.	
SI3	Using a smart cane makes me feel less dependent on people.	
ST1	I trust the reliability of information delivered by this system.	[17]
ST2	I trust this technology to keep personal information secure.	
ST3	The technology used looks trustworthy.	
BI1	I predict I would use a smart cane to manage my health information.	[24]
BI2	I can develop a habit to use a smart cane soon.	
BI3	In the future, I will often use a smart cane to manage my daily life.	
PU1	Using a smart cane will help me manage my health.	[51]
PU2	I believe that using a smart cane will make my daily life more convenient.	
PU3	Using a smart cane will improve my quality of life.	
PEOU1	Learning to use or operate the smart canes was easy for me.	[12]
PEOU2	I am easily proficient in using the smart canes.	
PEOU3	I think the interactive operation of the smart canes is clear and easy to understand.	

Note: PU = Perceived Usefulness; PEOU = Perceived Ease of Use; SI = Social Influence; ST = Safety Trust; ATT = Attitude; BI = Behavioral Intention; SE = Self-Efficacy.

**Table 3:** Data on indicators of confidence and convergent validity.

Construct	Item	Factor Loadings	Cronbach’s Alpha	CR	AVE
PU	PU1	0.783	0.835	0.838	0.633
	PU2	0.848			
	PU3	0.754			
PEOU	PEOU1	0.824	0.866	0.868	0.686
	PEOU2	0.847			
	PEOU3	0.815			
SI	SI1	0.743	0.817	0.817	0.599
	SI2	0.779			
	SI3	0.799			
ST	ST1	0.761	0.786	0.789	0.555
	ST2	0.779			
	ST3	0.692			
ATT	ATT1	0.779	0.835	0.835	0.627
	ATT2	0.800			
	ATT3	0.796			
BI	BI1	0.827	0.854	0.855	0.664
	BI2	0.835			
	BI3	0.781			
SE	SE1	0.786	0.875	0.878	0.706
	SE2	0.833			
	SE3	0.897			

Note: PU = Perceived Usefulness; PEOU = Perceived Ease of Use; SI = Social Influence; ST = Safety Trust; ATT = Attitude; BI = Behavioral Intention; SE = Self-Efficacy.

**Table 4:** Results of sample differentiation validity tests.

	PU	PEOU	SI	ST	ATT	BI
1. PU						
2. PEOU	0.673					
3. SI	0.299	0.400				
4. ST	0.620	0.584	0.421			
5. ATT	0.521	0.556	0.444	0.643		
6. BI	0.458	0.503	0.589	0.547	0.568	
7. SE	0.515	0.529	0.556	0.557	0.546	0.774

Note: PU = Perceived Usefulness; PEOU = Perceived Ease of Use; SI = Social Influence; ST = Safety Trust; ATT = Attitude; BI = Behavioral Intention; SE = Self-Efficacy.

**Table 5:** Results of direct paths hypothesis testing (N = 232).

Item	Hypothesis	Unstd.	S.E.	C.R.	*p*	Result
H1	ATT→BI	0.281	0.084	3.284	0.001	Supported
H2	SI→BI	0.322	0.075	4.273	***	Supported
H3.1	SI→ATT	0.187	0.085	2.142	0.032	Supported
H4	ST→BI	0.275	0.083	3.366	0.006	Supported
H5.1	ST→ATT	0.378	0.107	3.533	***	Supported
H6.1	SE→ATT	0.105	0.081	1.424	0.155	Not Supported
H7.1	PEOU→ATT	0.201	0.089	2.697	0.007	Supported
H8.1	PU→ATT	0.196	0.083	2.249	0.025	Supported
H9.1	PEOU→SE	0.224	0.072	3.102	0.002	Supported
H10.1	PU→SE	0.265	0.081	3.272	0.001	Supported

Note: Unstd. = Unstandardized Coefficient; S.E. = Standard Error; C.R. = Critical Ratio; *** *p* < 0.001.

**Table 6:** Mediating effect analysis.

Path Name	Path	Estimate	Lower	Upper	*p*
H3.2	SI→ATT→BI	0.312	−0.026	0.750	0.063
H5.2	ST→ATT→BI	0.378	0.025	0.914	0.036 *
H6.2	SE→ATT→BI	0.349	−0.035	0.677	0.071
H7.2	PEOU→ATT→BI	0.393	−0.027	0.861	0.062
H8.2	PU→ATT→BI	0.310	−0.174	0.727	0.166
H9.2	PEOU→SE→ATT	0.325	0.039	0.637	0.028 *
H9.3	PEOU→SE→ATT→BI	0.569	0.136	0.974	0.022 *
H10.2	PU→SE→ATT	0.365	0.015	0.720	0.043 *
H10.3	PU→SE→ATT→BI	0.609	0.142	0.985	0.018 *

Note: * *p* < 0.05.

## Data Availability

The raw data of this study cannot be publicly shared, as required by the Academic Ethics Committee of Guangdong University of Technology to protect participants’ privacy. However, anonymized summary data is available upon reasonable request to the corresponding author.
